# Hepatic Metabolic, Inflammatory, and Stress-Related Gene Expression in Growing Mice Consuming a Low Dose of *Trans*-10, *cis*-12-Conjugated Linoleic Acid

**DOI:** 10.1155/2012/571281

**Published:** 2012-09-02

**Authors:** Jing Li, Srikant Viswanadha, Juan J. Loor

**Affiliations:** ^1^Division of Nutritional Sciences, Mammalian NutriPhysioGenomics, Department of Animal Sciences, University of Illinois, 1207 West Gregory Drive, Urbana IL61801, USA; ^2^Dairy Science Department, Virginia Tech, Blacksburg, VA 24061, USA; ^3^Incozen Therapeutics Pvt. Ltd, “Spectrum” Discovery Zone, SP Biotech Park-Phase 1, Turkapally Mandal, Hyderabad 500078, India

## Abstract

Dietary *trans*-10, *cis*-12-conjugated linoleic acid (*trans*-10, *cis*-12-CLA) fed to obese and nonobese rodents reduces body fat but leads to greater liver mass due to steatosis. The molecular mechanisms accompanying such responses remain largely unknown. Our study investigated the effects of chronic low *trans*-10, *cis*-12-CLA supplementation on hepatic expression of 39 genes related to metabolism, inflammation, and stress in growing mice. Feeding a diet supplemented with 0.3% *trans*-10, *cis*-12-CLA (wt/wt basis) for 6 weeks increased liver mass and concentration of long-chain fatty acids (LCFAs) in liver, while adipose tissue mass decreased markedly. These changes were accompanied by greater expression of genes involved in LCFA uptake (*Cd36*), lipogenesis, and triacylglycerol synthesis (*Acaca*, *Gpam*, *Scd*, *Pck1*, *Plin2*). Expression of these genes was in line with upregulation of the lipogenic transcription factor *Srebf1*. Unlike previous studies where higher >0.50% of the diet) doses of *trans*-10, *cis*-12-CLA were fed, we found greater expression of genes associated with VLDL assembly/secretion (*Mttp*, *Cideb*), ketogenesis (*Hmgcs2*, *Bdh1*), and LCFA oxidation (*Acox1*, *Pdk4*) in response to *trans*-10, *cis*-12-CLA. Dietary CLA, however, did not affect inflammation- and stress-related genes. Results suggested that a chronic low dose of dietary CLA increases liver mass and lipid accumulation due to activation of lipogenesis and insufficient induction of LCFA oxidation and VLDL assembly/secretion.

## 1. Introduction

Conjugated linoleic acid (CLA) refers to a group of conjugated octadecadienoic acid isomers derived from linoleic acid (LA), a long-chain fatty acid (LCFA) that contains 18 carbons and 2 double bonds [1]. Antilipogenic effects of dietary CLA mixtures with a 1 : 1 ratio of *trans*-10, *cis*-12-CLA and *cis*-9, *trans*-11-CLA were initially observed in several lines of mice, and later work using purified isomer preparations clearly showed that *trans*-10, *cis*-12-CLA was responsible for delipidation [2, 3] (summarized in Table 1). Studies also have revealed unfavorable metabolic effects of *trans*-10, *cis*-12-CLA associated with an increased oxidative stress and inflammation [4] often coupled with hepatic steatosis [5–9]. As *trans*-10, *cis*-12-CLA supplementation reduces the amount of adipose tissue, several studies suggest that much of the increased liver mass may be explained by accumulation of lipid [10, 11] derived from breakdown of adipose tissue stores [12, 13].

Despite the clear deleterious effects observed in rodents, the European Food Safety authority after an evaluation of the available data concluded that consumption of 3.5 g CLA (or 4.5 g product/d) from a commercial product containing equal proportions of *trans*-10, *cis*-12-CLA and *cis*-9, *trans*-11-CLA was not deleterious to health [14]. However, at the molecular level, hepatic steatosis in rodents due to *trans*-10, *cis*-12-CLA at ≥0.5% of the diet has been linked to alterations in the expression of genes associated with energy expenditure, apoptosis, fatty acid oxidation, lipolysis, differentiation, and lipogenesis [1, 15]. Therefore, it is likely that sustained supplementation of the above amount, which is much greater than the 3.1 mg CLA/kg body weight estimated previously [16], has the potential to alter tissue expression of genes in pathways of metabolism, inflammation, and stress as has been shown in rodent studies.

To our knowledge there are no published studies dealing with the hepatic transcriptome in response to low chronic dietary *trans*-10, *cis*-12-CLA supplementation. Our general hypothesis was that long-term increases in liver mass in response to dietary *trans*-10, *cis*-12-CLA at low doses would be associated with alterations in mRNA abundance of lipogenic, fatty acid oxidation, and inflammatory genes. Specific objectives were to measure mRNA abundance of genes encoding proteins required for LCFA uptake, LCFA oxidation, de novo LCFA synthesis, lipolysis, lipogenesis, ketogenesis, inflammation, stress response, transcriptional regulation of lipogenesis, and lipid droplet formation.

## 2. Materials and Methods

### 2.1. Animals, Diets, Sampling, and Fatty Acid Analysis

All experiments were approved by the Institutional Animal Care and Use Committee (IACUC) at Virginia Tech. Male CD-1 mice from Harlan (Madison, Wis, USA), 3 to 4 weeks of age, were divided into two experimental groups of ten mice each. Mice were housed individually with access to food and water at all times. A 12-h light/12-h dark cycle was maintained throughout the study. For 7 d before the start of the study, all mice were fed Harlan Teklad (Global Rodent Diet, 18% protein, 5% fat; Harlan, Madison, WI, USA) with 3% (w/w) high-oleic sunflower oil added. On day 1 of the study, mice were randomly assigned to receive diets containing 3.0% high-oleic sunflower (control) or 2.70% high-oleic sunflower oil + 0.30% *trans*-10, *cis*-12-CLA. The free FA form of *trans*-10, *cis*-12-CLA (>95% purity) was obtained from Natural Lipids (Hovdebygda, Norway). Mice were fed daily at 1600 hours. At the end of weeks 2 and 6, five mice per dietary treatment were anesthetized using Metofane (Pitman-Moore, Inc., Washington Crossing, NJ, USA) prior to cervical dislocation. The liver was removed, rinsed with diethylpyrocarbonate (Sigma, St. Louis, MO, USA) in distilled water (1 : 1000, v/v), and weighed. A portion of liver was frozen in liquid nitrogen and later stored at −80°C prior to RNA extraction and gene expression analysis.

Total LCFA in liver were extracted as described previously [17]. Undecenoic acid (Nu-Check Prep, Inc., Elysian, Min, USA), added to extracted lipids prior to formation of fatty acid methyl esters (FAME) [18], served as an internal standard. Using an Agilent 6890N gas chromatograph equipped with a flame ionization detector (Agilent Technologies, Palo Alto, Calif, USA), FAME were separated with a 100 m × 0.25 mm i.d. (0.2 *μ*m film thickness) CP-Sil 88 column (Varian, Lake Forest, Calif, USA) protected by a 2.5 m × 0.25 mm i.d. fused silica retention gap. Ultrapure hydrogen at constant pressure was the carrier gas. Pure methyl ester standards (Nu-Check Prep, Inc., Pa, USA) were used for identification and quantification of sample FA [17].

### 2.2. Additional Materials and Methods

Specific details of RNA extraction and quantitative real-time RT-PCR (qPCR) are reported in additional file 1 (supplementary material). qPCR performance, primer features, and sequencing are reported in Tables S1–S4 (additional file 1), (see Supplementary material available online at doi:10.1155/2012/571281). Results of internal control gene (ICG) evaluation are shown in Figure S1 (additional file 1).

### 2.3. Statistical Analysis

Data were analyzed as a completely randomized design with a factorial arrangement of treatments using the MIXED procedure of SAS (Windows version 9.1, Cary, NC, USA). Normalized gene expression data using the geometric mean of the top 4 most-stable ICG (Figure S1, additional file 1) were log-transformed prior to statistical analysis. The model for gene expression and liver LCFA profiles included the fixed effects of dietary treatment (0% or 0.30% CLA), time (week 2 and 6), and the interaction of dietary treatment × time. Mouse within dietary treatment × time was the random effect. Least-squares means ± standard error of the means (SEM), (combined observations for weeks 2 and 6) are presented in the tables.

## 3. Results

### 3.1. Tissue Weight

There was no difference in final live body weight between treatments, and the average was 37.1 g (Table 2). Liver weight was ~19% greater in mice fed CLA and averaged 2.36 g versus 1.99 g in controls. In contrast, the weight of epididymal adipose tissue was 23% lower in CLA-fed mice compared with controls, that is, 0.70 g versus 0.91 g. Carcass weight was lower in CLA-fed mice compared with controls, averaging 12.8 g versus 14 g.

### 3.2. Long-Chain Fatty Acid Concentration in Liver

The total concentration of LCFA in mice fed CLA increased by ~42% and averaged 60.3 *μ*g/mg dry liver tissue versus 42.5 *μ*g/mg (Table 3). There was an overall increase in LCFA concentration over time regardless of treatment, with concentration averaging 41 *μ*g/mg at week 2 and increasing to 61.7 *μ*g/mg at week 6. The concentration of 18 : 0 was lower in mice fed CLA (14% versus 19.6%), and it decreased markedly regardless of treatment between week 2 and 6 (20.9% to 12.7%). The concentration of *cis*9-18:1, the product of 18 : 0 desaturation, was greater in mice fed CLA (24.4% versus 20.1%) and also decreased between week 2 and 6 (24.4% to 20.2%). As expected, the concentration of *trans*-10, *cis*-12-CLA was greater in mice fed CLA (0.30% versus <0.00%) and also the concentration of *trans*-10-18:1 in those mice was greater (0.05% versus 0.02%) relative to controls. Overall, the concentration of *cis*-9, *cis*-12-18:2, 20:4n-6, and 22:6n-3 increased between week 2 and 6 regardless of treatment; whereas concentration of *cis*-9,*cis*-12,*cis*-15-18:3 decreased over the same time frame regardless of treatment.

### 3.3. Markers of Fatty Acid Uptake in Liver

The expression of *Cd36* was greater (CLA = 1.11 versus control = −1.47; *P* = 0.001; Table 4) with* trans*-10, *cis*-12-CLA and there was no change due to treatment for *Slc27a2* expression.

### 3.4. Lipogenesis and TG Synthesis

We observed that *Acaca* (CLA = 0.72 versus control = 0.22) and *Scd* (CLA = 0.05 versus control = −0.92) expression was upregulated in response to *trans*-10, *cis*-12-CLA. Among the genes associated with TG synthesis, we observed upregulation of *Gpam *in mice fed *trans*-10, *cis*-12-CLA (CLA = 0.44 versus control = −0.31, but the expression of *Dgat1* and *Dgat2* did not differ. Dietary CLA upregulated *Pck1* (CLA = −0.29 versus control = −1.74) and also the lipid droplet-associated protein *Plin2* (CLA = 0.40 versus control = −0.43).

### 3.5. Transcription Regulation and Lipogenesis and TG Synthesis

Although the expression of glucose-regulated transcription factor *Chrebp* did not differ between treatments, we observed that *Srebf1* expression in mice fed *trans*-10, *cis*-12-CLA was upregulated (CLA = 0.14 versus control = −0.22).

### 3.6. VLDL Assembly and Secretion

We observed significant upregulation of *Mttp* (CLA = 0.14 versus control = −0.37) and *Cideb* (CLA = −0.04 versus control = −1.13) in mice fed *trans*-10, *cis*-12-CLA, but expression of *Apob* did not differ.

### 3.7. Carbohydrate Metabolism

We observed no treatment effects in the expression of genes associated with glucose transport (*Slc2a1*), glycolysis (*Gck*), and gluconeogenesis (*Pc*).

### 3.8. Lipolysis

We observed no treatment effects in the expression of the lipolytic genes *Lipc* and *Pnpla2*.

### 3.9. Ppar*α*, Lipid Catabolism, and Energy Metabolism

We did not observe differences in expression of *Ppara* but expression of its targets *Pdk4 *(CLA = 0.47 versus control = −1.85), *Acox1 *(CLA = 0.13 versus control = −0.22), and *Ucp2* (CLA = 0.15 versus control = −0.31) was upregulated (Table 5). Another well-established *Ppara *target such *Cpt1a *did not differ due to CLA but the expression of the ketogenic regulator *Hmgcs2* (CLA = 0.13 versus control = −0.16 and enzyme *Bdh1* (CLA = 0.13 versus control = −0.20) was upregulated with CLA. 

### 3.10. Inflammation and Stress Response

The expression of one gene associated with inflammation (*Saa1*) and 8 genes associated with intracellular stress (*Angptl3, Angptl4, Atf6, Ddit3, Eif2ak3, Fgfr2, Hspa1b, Xbp1*) did not differ due to treatments (Table 6).

## 4. Discussion

Several studies have shown that *trans*-10, *cis*-12-CLA at ≥0.5% of the diet reduced adipose mass and increased liver mass (summarized in Table 1). In the present study, we fed a low dose of *trans*-10, *cis*-12-CLA that may be considered more physiologically relevant, compared with previous studies, from a nutritional standpoint to examine any potential links between adipose mass loss, liver mass, and expression of genes related to various aspects of lipid metabolism. Hepatic enlargement has been associated with a 3- to 7-fold increase in TG content (Table 1), and we also observed an increase in liver mass and accumulation of LCFA (Tables 2 and 3). 

### 4.1. *Trans*-10, *cis*-12-CLA Increased Markers of Fatty Acid Uptake in Liver

Our results are in agreement with previous data showing two-fold greater hepatic LCFA content [7] and lower serum LCFA concentration in mice fed *trans*-10, *cis*-12-CLA [13]. Therefore, upregulation of *Cd36* expression with *trans*-10, *cis*-12-CLA allows for adipose tissue-derived-LCFA uptake into hepatocytes and, in fact, may represent a mechanism unique to this isomer because it has been shown *in vitro* that uptake of* trans*-10, *cis*-12-CLA in rat hepatocytes was 3X greater than for oleate [19]. Furthermore, a previous study showed not only a significant increase in hepatic *Cd36* expression in mice fed *trans*-10, *cis*-12-CLA but also a significant association between *Cd36* expression and the degree of hepatic steatosis [20]. Therefore, upregulation of *Cd36* at low dietary *trans*-10, *cis*-12-CLA is part of the mechanism leading to increased hepatic fatty acid uptake and hepatic steatosis.

### 4.2. *Trans*-10, *cis*-12-CLA Stimulates Lipogenesis and TG Synthesis in Liver

Several authors have concluded that de novo fatty acid synthesis via acetyl-CoA carboxylase (Acaca) and fatty acid synthase (Fasn) may play a role in the onset of hepatic steatosis related to CLA supplementation [21, 22]. The upregulation of *Acaca* with CLA suggested that the liver was generating more malonyl-CoA which was then used for palmitic acid synthesis by the multifunctional enzyme Fasn. The numerically greater concentration of 16 : 0 in CLA-fed mice (24.1% versus 22.5%) provides some support for such an effect. Ashwell et al. [15] and Kelley et al. [7] observed greater 16 : 0 in liver tissue of obese mice fed *trans*-10, *cis*-12-CLA than those fed a linoleic acid or corn oil.

Stearoyl-CoA desaturase (Scd) is the rate-limiting enzyme involved in the synthesis of mono-unsaturated fatty acids (*cis*-9-16 : 1 or *cis*-9-18 : 1) from saturated fatty acids (16 : 0 or 18 : 0) [23]. Although not in the glycerol-3-phosphate pathway of TG synthesis, Scd plays an important role in the synthesis of TG [23]. The expression of the mouse *Scd* gene is regulated primarily at the level of transcription [24], and its expression in adipose tissue decreases as the *trans*-10, *cis*-12-CLA content of the diet increases [6]. The upregulation of *Scd* we observed with CLA confirmed results from Guillén et al. [20] in mice. Furthermore, the upregulation of *Scd* in our study correlated with the lower concentration of 18 : 0 (substrate) and greater concentration of *cis*9–18 : 1 (main product), (Table 3). Because a large number of studies have shown that *trans*-10, *cis*-12-CLA reduces body fat mass considerably, the upregulation of hepatic *Scd* mRNA due to feeding *trans*-10, *cis*-12-CLA may be a response to handle the influx of 16 : 0 and 18 : 0 derived from adipose tissue lipolysis.

The initial reaction in the pathway of TG synthesis is catalyzed by glycerol-3-phosphate acyltransferase (Gpam) which resides in the outer mitochondrial membrane [25] and it is the first committed step in the synthesis of TG [25] via the glycerol phosphate pathway. In addition, Gpam is an enzyme that can determine the fate of LCFA between *β* oxidation or glycerolipid synthesis [26]. Subsequent steps of TG synthesis are regulated by Dgat1 and Dgat2, while Pck1 provides a source of glycerol-3-phosphate during glyceroneogenesis [27]. Perilipin (Plin) proteins are vital for cytoplasmic lipid droplet (LD) formation [28]. In isolated hepatocytes, as well as livers from mice and humans, Plin2 levels are proportional to hepatic lipid content [29]. 

Our results for *Gpam* expression are contrary to a previous study which found that *trans*-10, *cis*-12-CLA did not change the hepatic expression of *Gpam *in mice [15]. The concerted upregulation of *Acaca*,* Gpam*,* Pck1*, and *Plin2* could be taken as a mechanistic response to promote biosynthesis of TG and lipid droplet formation, for example, the upregulation of *Acaca* stimulates de novo FA synthesis, thus, generating more precursors (i.e., feed-forward effect) for TG synthesis leading to stimulation of both *Gpam* and *Pck1* expression. In addition, Plin protects lipid droplets from cAMP-mediated lipolysis [30], thus, an increase in *Plin2* expression represents attenuation of lipolytic enzyme-mediated TG breakdown within the lipid droplet. Those scenarios also would allow for blood LCFA derived from adipose tissue lipolysis to be esterified into TG within liver. Our results support the hypothesis from studies feeding much higher doses of *trans*-10, *cis*-12-CLA that hepatic lipogenesis and esterification are primary factors responsible for liver TG accumulation [5, 21, 22]. 

### 4.3. *Trans*-10, *cis*-12-CLA Increases VLDL Assembly and Secretion

In the liver, synthesized TG is either stored in cytoplasmic droplets or secreted as VLDL particles [31]. Most de novo-synthesized TG is stored in cytosolic TG pools and a smaller portion is secreted in the form of VLDL. Apo-protein B 100 (Apob) is the key component whose rate of synthesis in the rough endoplasmic reticulum controls the overall rate of VLDL production [32]. Lipid components are added to apoprotein B by microsomal TG transfer protein (Mttp). Because Mttp catalyzes the transfer of lipids to the ApoB molecule, Mttp may play a crucial role in the overall assembly and secretion of VLDL in the liver. In addition to these proteins, recent work has uncovered a potentially important function for the cell death-inducing DFF45-like effector (Cide) isoform b (Cideb) protein in the process of VLDL synthesis, for example, liver of Cideb-null mice had higher levels of cytosolic TG accompanied by lower levels of VLDL secretion [33]. Cideb is localized to smooth ER and LD and interacts physically with ApoB [33]. 

Despite the well-described accumulation of TG in the liver due to CLA (Table 1), which leads to greater tissue mass and greater LCFA (Tables 2 and 3), our results suggested that the VLDL assembly/secretion mechanism in mice fed *trans*-10, *cis*-12-CLA was not impaired and may have actually been enhanced via the upregulation of hepatic *Mttp* and *Cideb*. It was reported previously that in *trans*-10, *cis*-12-CLA-fed mice the VLDL secretion rate was increased; whereas, the plasma TG concentration was decreased [12]. Taken together, results suggest that the enhanced VLDL secretion rate is insufficient to eliminate excess fatty acids (Table 2) entering the esterification and VLDL assembly/secretion pathways resulted from increased fatty acid uptake, which would account for part of the fat deposited within liver cells. Our finding with *Mttp* is in agreement with recent kinetic studies showing that the actual amount of hepatic TG-rich lipoprotein secretion rate during nonalcoholic fatty liver disease is actually increased, but it is inadequate to match the increased TG synthesis in the liver [34]. The fact that* Cideb* requires both its ApoB-binding and LD association domains to allow for secretion of TG-enriched VLDL particles seems to support our findings, that is, Cideb also is required to promote the formation of TG-enriched VLDL particles [33]. 

### 4.4. Transcription Regulation and Lipogenesis and TG Synthesis

Sterol regulatory element binding transcription factor 1 (Srebf1) is the key transcription factor regulating hepatic lipogenesis in rodents [35]. Its upregulation with CLA is in agreement with the data on lipogenic genes from the current study as well as previous reports on mice fed higher levels of CLA-mixtures [5]. It is unclear, however, what signal might have triggered upregulation of *Srebf1* in our study, but insulin may have played a role. In freshly isolated hepatocytes, *Srebf1* mRNA was activated by insulin [36]. Thus, if feeding CLA in our study resulted in greater blood insulin, as previously reported [5, 9], it would explain the upregulation of *Srebf1*. The coordinated greater expression of the *Srebf1* target genes *Acaca*, *Scd*, and *Gpam* in mice fed *trans*-10, *cis*-12-CLA suggested that the liver was generating more palmitic acid and oleic acid (see Table 3) and esterifying it into TG. Collectively, these results indicated that low dietary *trans*-10, *cis*-12-CLA stimulated Srebf1 leading to an overall increase in fat accumulation in liver.

### 4.5. Ppar*α* and Lipid Catabolism due to *trans*-10, *cis*-12 CLA

Isomers of CLA have some structural features similar to peroxisome proliferators and, importantly, the physiological responses observed in mice fed high doses of CLA (e.g., reduced body mass, hepatic lipid accumulation, hypolipidemia) are characteristic of this group of chemicals [37]. The ligand-activated transcription factor peroxisome proliferator-activated receptor *α* (Ppara) modulates lipid catabolism partly via activation of *Lpl* [37], *Acox1* [38], *Cpt1a*, thioesterases, fatty acid binding protein, peroxisomal and mitochondrial *β* oxidation enzymes, microsomal *ω*-oxidizing enzymes, and apolipoproteins [35]. *Trans*-10, *cis*-12-CLA has been reported to be a potent ligand of *Ppara* [37] and, despite the lack of change in Ppara expression, the upregulation of several of its target genes playing important roles in lipid metabolism (*Cd36*, *Pdk4*, *Acox1*, *Ucp2*) provided indirect evidence that *trans*-10, *cis*-12-CLA might act through Ppara. 

Because a decrease in fatty acid oxidation may cause fat accumulation in liver [13], it is intuitive that the mRNA expression of the enzymes related to fatty acid oxidation may be downregulated during liver lipidosis. However, recent investigations of *trans*-10, *cis*-12-CLA effects on fatty acid oxidation are controversial. Rasooly et al. [39] found that *trans*-10, *cis*-12-CLA supplementation decreased the expression of *Cpt1a* (−77%), *Acox1* (−50%), and *Ppar*α** (−65%). However, in the study of Gruffat et al. [19] liver tissue samples of rats incubated with a fatty acid mixture (representative of circulating LCFA) and *trans*-10, *cis*-12-CLA or oleate, they found that the rate of CLA, isomer oxidation was 2X higher than that of oleate. Furthermore, expression of hepatic fatty acid oxidation genes in mice also increased in three studies with mixtures of CLA isomers [21, 22] and in one study with *trans*-10, *cis*-12-CLA [13].

Cpt1a is considered the rate-limiting enzyme for mitochondrial *β* oxidation [39]. Degrace et al. [13] found an increase in the activity of hepatic Cpt1a in mice supplemented with *trans*-10, *cis*-12-CLA accompanied by an increase in Acox1 activity and gene expression. The lack of change in *Cpt1a* that we observed with CLA may have been associated with the upregulation of *Acaca* due to CLA, that is, greater flux through Acaca would have generated malonyl-CoA which is a potent inhibitor of Cpt1a [32]. Because mice were fed over a 6-week period in the current study, it is possible that adaptive changes occurred wherein the increase in *Cpt1a* was prevented by the elevated levels of malonyl-CoA. As a consequence of this effect on *Cpt1a*, more LCFA would have been available for esterification into TG and accumulation in the cytosol.

In addition to mitochondrial oxidation, peroxisomes also play an important role in LCFA oxidation [32]. Acyl-CoA oxidase 1 (Acox1) catalyzes the first and rate-limiting enzyme of the peroxisomal fatty acid *β* oxidation pathway [32], and mice lacking *Acox1* developed severe steatohepatitis [40]. The upregulation of *Acox1* with CLA that we observed suggested an increase in fatty acid *β* oxidation in peroxisomes. The upregulation of *Acox1* might have been due to the relatively lower amount of dietary *trans*-10, *cis*-12-CLA supplementation compared with previous studies [5, 9–11, 15, 41]. Furthermore, Acox1 is not affected by malonyl-CoA as Cpt1a and, as such, would represent a mechanism for excess LCFA (e.g., incoming LCFA from adipose catabolism) catabolism to continue on the face of greater lipogenic rates. 

Regulation of the pyruvate dehydrogenase complex (PDC) is an important step in fuel selection of energy utilization in animals during different nutritional and hormonal states as the modulation of PDC activity impacts fatty acid as well as pyruvate and glucose metabolism [42]. Phosphorylation of PDC via pyruvate dehydrogenase kinase (Pdk) inhibits its activity [42], thereby reducing the conversion of pyruvate to acetyl-CoA and sparing glucose oxidation in favor of LCFA. The abundance of the *Pdk4* isoform, which is highly expressed in liver, heart, and skeletal muscle, is transcriptionally controlled by Pppara [42]. The upregulation of *Pdk4 *with CLA provided evidence of reduced glucose oxidation and supports the view that peroxisomal oxidation of LCFA might have been increased. Because expression of *Pdk4* is induced by high-fat diets and LCFA [43], the greater *Pdk4* expression was likely a consequence of *trans*-10, *cis*-12-CLA intake and its catabolic effect on adipose tissue (Tables 1 and 2).

Under conditions of increased fatty acid uptake and oxidation the liver produces large amounts of acetoacetate and D-3-hydroxybutyrate during the process of ketogenesis [32]. Ketogenesis is controlled indirectly by Cpt1a (i.e., via provision of acetyl-CoA) and directly by the activity of the mitochondrial key regulatory enzyme 3-hydroxy-3-methylglutaryl-CoA synthase 2 (Hmgcs2), which is a Ppara target [44]. The enzyme 3-hydroxybutyrate dehydrogenase 1 (Bdh1) plays a key role in redox balance and energy metabolism since in the presence of NADH, the hepatic Bdh transforms acetoacetate into D-3-hydroxybutyrate, which is then transported through the blood stream to peripheral tissues, for example, brain, heart, and kidney [45]. Our results support previous findings of greater hepatic *Hmgcs2* and *Bdh1* expression in mice fed *trans*-10, *cis*-12-CLA. Thus, influx of LCFA from adipose lipolysis not only enhanced peroxisomal oxidation but also resulted in increased ketogenesis, which was in agreement with a previous study [46]. Together, data suggested that there was a stimulation of ketone body production in liver due to feeding CLA.

### 4.6. *Trans*-10, *cis*-12-CLA Changes Energy Metabolism in Liver

Uncoupling proteins (Ucp) belong to a family of mitochondrial anion carriers and are present in the mitochondrial inner membrane [47]. Ucp dissipate the proton gradient by allowing the reentry of protons into the mitochondrial matrix during oxidative ATP generation, resulting in the uncoupling of the respiratory chain and heat production [48]. *In vivo* studies indicated that physiological and pathological elevation of blood LCFA resulting from fasting [49] or high-fat diets [50] induced upregulation of *Ucp2*. Peters et al. [51] demonstrated that dietary CLA increases *Ucp2* expression in murine liver, which we confirmed. The uncoupling process might have served as a counterregulatory mechanism to lower cellular ATP levels and decreased metabolic efficiency, thereby serving as a mechanism to reduce fat accumulation in the long term [52].

### 4.7. *Trans*-10, *cis*-12-CLA, and the Stress Response

According to the complex mechanisms proposed by Jaudszus et al. [6], the antiadipogenic effect of *trans*-10, *cis*-12-CLA in adipose tissue may be primarily the consequence of proapoptotic and proinflammatory responses, including the nuclear factor *κ*B- (NF*κ*B-) dependent production of tumor necrosis factor-*α*, interleukin-6, and interleukin-8 [8, 9]. The lack of alteration in the expression of several genes associated with inflammation or stress response was likely related to the relatively low dosage of *trans*-10, *cis*-12-CLA that was fed.

In conclusion, our study showed that even at one of the lowest dietary doses of *trans*-10, *cis*-12-CLA studied to date, the coordinated upregulation of genes associated with fatty acid uptake, TG synthesis, and lipid droplet formation as well as insufficient induction of VLDL assembly/secretion contribute to enhanced TG accumulation and greater liver mass. Impaired mitochondrial *β*-oxidation did not appear to be a factor contributing to hepatic steatosis, partly due to a greater contribution of the peroxisomal pathway.

## Supplementary Material

The supplemental file contains information on methods used for extraction of RNA and quantitative PCR, primer design and testing, evaluation of suitable internal control genes (ICG) for normalization, quantitative PCR performance (Table S1), and primer sequences (Table S2) and sequencing results (Table S3 and S4). Figure S1 depicts the results from analysis of ICG using geNorm software.

## Figures and Tables

**Table 1:** Summary of the changes in liver mass, liver TG, and adipose tissue mass observed in previous mouse studies feeding different levels of *trans*-10, *cis*-12-CLA compared with feeding linoleic acid (LA) or oleic acid (as canola oil).

Reference	Dose	Duration	*trans*-10, *cis*-12 CLA response^2^		
			Liver mass	Liver TG/fat	Adipose mass
Clément et al. (2002) [5]	0.4%	4 weeks	3.1 fold ↑	N.A^1^	80% ↓
Halade et al. (2009) [53]	0.5%	0.5 year	50% ↑	N.A	73% ↓
Degrace et al. (2003) [12]	10 g	4 weeks	60% ↑	655% ↑	83% ↓
Kelley et al. (2004) [7]	0.5%	8 weeks	N.A	5 fold↑	N.A
House et al. (2005) [10]	1%	2 weeks	33% ↑	61% ↑	58% ↓
Warren et al. (2003) [54]	0.5%	8 weeks	98% ↑	427% ↑	59% ↓
Degrace et al. (2004) [13]	1%	4 weeks	80% ↑	646% ↑	85% ↓

^1^Not available.

^2^Percentage = [Wt of CLA-fed Mice − Wt of LA or canola oil-fed Mice]/[Wt of LA or CO-fed Mice]×100.

**Table 2:** Live body weight and weights of selected tissues and carcass in mice fed *trans*-10, *cis*-12-CLA (CLA).

	Treatment		SEM	*P*
	Control	CLA		
Body weight (g)	38.6	35.6	1.6	0.24
Liver (g)	1.99^b^	2.36^a^	0.12	0.04
Epididymal adipose (g)	0.91^a^	0.70^b^	0.04	0.01
Carcass (g)	14.0^a^	12.8^b^	0.32	0.01

^a,b^Means with different superscripts differ (*P* ≤ 0.05).

SEM: the largest standard error of the mean is shown.

**Table 3:** Long-chain fatty acid concentrations in liver from mice fed control or the control supplemented with *trans*-10, *cis*-12-CLA (CLA). There were few significant interactions of treatment × time. Therefore, the results are presented according to main effects (treatment and time).

Item	Treatment		Time		SEM
	Control	CLA	Week 2	Week 6
Total fatty acids, *μ*g/mg dry liver	42.5^b^	60.3^a^	41.0^b^	61.7^a^	4.5
16: 0	22.5	24.1	28.6^a^	17.9^b^	1.2
18: 0^3^	19.6^a^	14.0^b^	20.9^a^	12.7^b^	1.1
*cis*-9-18: 1	20.1^b^	24.4^a^	24.4^a^	20.2^b^	1.3
*trans*-10-18: 1	0.02^b^	0.05^a^	0.05^a^	0.02^b^	0.01
*trans*-11-18: 1	0.07	0.06	0.07	0.05	0.01
*cis*-9,*cis*-12-18: 2	18.0	19.2	13.5^b^	23.7^a^	1.4
*cis*-9,*cis*-12,*cis*-15-18: 3	0.60	0.56	0.68^a^	0.48^b^	0.07
CLA isomers					
*cis*-9,*trans*-11-18: 2	0.01	0.03	0.03	0.02	0.01
*trans*-10,*cis*-12-18: 2^1^	<0.00^b^	0.30^a^	0.11^b^	0.19^a^	0.02
Total^1^	0.13^b^	0.46^a^	0.29	0.30	0.02
20: 4n-6	8.3	6.1	3.0^b^	11.4^a^	0.9
22: 6n-3	4.1	3.4	1.0^b^	6.5^a^	0.6

Total 20-carbon	14.2	11.4	5.3^b^	20.2^a^	1.5

^a,b^Means within each main effect (treatment and time) with different superscripts differ (*P* ≤ 0.05).

SEM: the largest standard error of the mean is shown.

^1^Significant treatment × time.

**Table 4:** Hepatic expression of genes related to LCFA uptake, lipogenesis, TG synthesis, lipoprotein synthesis, and transcription regulation in mice fed control or the control supplemented with *trans*-10, *cis*-12-CLA. There were no significant interactions of treatment × time. Therefore, the results are presented according to main effects (treatment and time).

Gene	Treatment		Time		SEM
	Control	CLA	Week 2	Week 6
Fatty acid uptake					
*Cd36*	−1.47^a^	1.11^b^	−0.01	−0.35	0.55
*Slc27a2*	−0.23	−0.04	−0.07	−0.20	0.18
De novo synthesis and desaturation					
*Acaca*	0.22^a^	0.72^b^	0.42	0.51	0.11
*Scd*	−0.92^a^	0.05^b^	−0.50	−0.37	0.19
TG and lipid droplet synthesis					
*Dgat1*	−0.02	0.30	0.23	0.05	0.17
*Dgat2*	0.03	0.25	0.26	0.02	0.15
*Gpam*	−0.31^a^	0.44^b^	0.13	0.004	0.17
*Pck1*	−1.74^a^	−0.29^b^	−0.80	−1.24	0.62
*Plin2*	−0.43^a^	0.40^b^	−0.01	−0.02	0.18
Transcription regulation					
*Chrebp*	−0.22	−0.17	−0.19	−0.19	0.08
*Srebf1*	−0.22^a^	0.14^ b^	0.04	−0.11	0.11
VLDL synthesis					
*Apob*	−0.33	−0.05	−0.09	−0.29	0.19
*Cideb*	−1.13^a^	−0.04^b^	−0.35	−0.81	0.50
*Mttp*	−0.37^a^	0.14^b^	0.002	−0.22	0.17
Carbohydrate Metabolism					
*Gck*	−0.45	−0.49	−0.31	−0.63	0.28
*Pc*	−0.31	−0.08	−0.08	−0.32	0.28
*Slc2a1*	−0.07	0.21	0.18	−0.04	0.14
Lipolysis					
*Lipc*	−0.95	−0.20	−0.15	−1.00	0.54
*Pnpla2*	−0.04	0.14	0.24	−0.13	0.24

^a,b^Means within each main effect (treatment and time) with different superscripts differ (*P* ≤ 0.05).

SEM: the largest standard error of the mean is shown.

**Table 5:** Hepatic expression of genes related to lipid catabolism in mice fed control or the control supplemented with *trans*-10, *cis*-12-CLA (CLA). There were no significant interactions of treatment × time. Therefore, the results are presented according to main effects (treatment and time).

Gene	Treatment		Time		SEM
	Control	CLA	Week 2	Week 6
Fatty acid oxidation and energy metabolism					
*Aacs*	−0.21	−0.29	−0.26	−0.25	0.31
*Acox1*	−0.22^a^	0.13^b^	−0.08	−0.003	0.14
*Bdh1*	−0.20^a^	0.13^b^	0.06	−0.12	0.12
*Cpt1a*	−0.43	−0.32	−0.36	−0.39	0.14
*Insig1*	−0.79	0.35	−0.23	−0.20	0.45
*Mlycd*	0.001	−0.17	−0.07	−0.11	0.10
*Pdk4*	−1.85^a^	0.47^b^	−0.81	−0.57	0.50
*Ucp2*	−0.31^a^	0.15^b^	−0.04	−0.11	0.10
*Hmgcs2*	−0.16^a^	0.13^b^	0.10^a^	−0.13^b^	0.11
Transcription regulation					
*Ppar* **α**	−0.33	−0.19	−0.23	−0.30	0.12

^a,b^Means within each main effect (treatment and time) with different superscripts differ (*P* ≤ 0.05).

SEM: the largest standard error of the mean is shown.

**Table 6:** Hepatic expression of genes related to inflammation and cellular stress in mice fed control or the control supplemented with *trans*-10, *cis*-12-CLA (CLA). There were no significant interactions of treatment × time. Therefore, the results are presented according to main effects (treatment and time).

Gene	Treatment		Time		SEM
	Control	CLA	Week 2	Week 6
Inflammation					
*Saa1*	−0.50	−0.21	−0.17	−0.54	0.31
Stress response					
*Angptl3*	−0.54	0.07	−0.26	−0.21	0.42
*Angptl4*	0.01	−0.29	−0.53	0.25	0.35
*Atf6*	−0.27	0.17	0.16	−0.26	0.46
*Ddit3*	−0.25	0.37	−0.11	0.24	0.30
*Eif2ak3*	−0.55	−0.10	−0.32	−0.32	0.49
*Fgfr2*	−0.09	0.19	−0.58	0.68	0.49
*Hspa1b*	−1.04	−0.54	−0.91	−0.67	0.62
*Xbp1*	−1.15	−0.55	−0.46	−1.23	0.63

^a,b^Means within each main effect (treatment and time) with different superscripts differ (*P* ≤ 0.05).

SEM: the largest standard error of the mean is shown.
