# Supplementary material for: Hepatic Metabolic, Inflammatory, and Stress-Related Gene Expression in Growing Mice Consuming a Low Dose of Trans-10, cis-12-Conjugated Linoleic Acid
Source: J Lipids. 2012 Sep 2;2012:571281. doi: 10.1155/2012/571281 (PMC3438780; doi:10.1155/2012/571281)
Supplement: Supplementary file 1 — The supplemental file contains information on methods used for extraction of RNA and quantitative PCR, primer design and testing, evaluation of suitable internal control genes (ICG) for normalization, quantitative PCR performance (Table S1), and primer sequences (Table S2) and sequencing results (Table S3 and S4). Figure S1 depicts the results from analysis of ICG using geNorm software. [file 571281.f1.doc]

**Extraction and analysis of RNA and semiquantification by RT-PCR**

Liver RNA was extracted using TRI REAGENT® (MRC, Cincinnati, OH) and according to the manufacturer’s instructions. Genomic DNA was removed from RNA with DNase (Qiagen) using RNeasy Mini Kit columns (Qiagen, Germany). RNA concentration was measured with a NanoDrop ND-1000 spectrophotometer (NanoDrop Technologies). The purity of RNA (A_260_/A_280_) for all samples was above 1.8. RNA quality was assessed using a 2100 Bioanalyzer (Agilent Technologies). A portion of assessed RNA was diluted to 100 ng/μl using DNase-RNase free water prior to reverse transcriptase.

Each cDNA was synthesized by RT-PCR using 100 ng RNA, 1 μl dT18 (Operon Biotechnologies, AL), 1 μl 10 mmol/L dNTP mix (Invitrogen Corp., CA), 1 μl random primers (Invitrogen Corp., CA), and 10 μl DNase/RNase free water. This mixture was incubated at 65℃ for 5 min and kept on ice for 3 min. A total of 6 μl of Master Mix composed of 4.5 μl 5X First-Strand Buffer, 1 μl 0.1 M DTT, 0.25 μl (50u) of SuperScript^TM^ III RT (Invitrogen Corp., CA), and 0.25 μl of RNase Inhibitor (10U, Promega, WI) was added. The reaction was performed in an Eppendorf Mastercycler® Gradient using the following temperature program: 25℃ for 5 min, 50℃ for 60 min, and 70℃ for 15 min. cDNA was the diluted 1:4 with DNase/RNase free water.

Quantitative PCR (qPCR) was performed using 4 μl of diluted cDNA combined with 6 μl of a mixture composed of 5 μl 1× SYBR Green master mix (Applied Biosystems, CA), 0.4 μl each of 10 μM forward and reverse primers, and 0.2 μl DNase/RNase free water in a MicroAmp^TM^ Optical 384-Well Reaction Plate (Applied Biosystems, CA). Each sample was run in triplicate and a 5 point relative standard curve plus the non-template control (NTC) were used (User Bulletin #2, Applied Biosystems, CA). The reactions were performed in an ABI Prism 7900 HT SDS instrument (Applied Biosystems, CA) using the following conditions: 2 min at 50℃, 10 min at 95℃, 40 cycles of 15 s at 95℃ (denaturation), and 1 min at 60℃ (annealing + extension). The presence of a single PCR product was verified by the dissociation protocol using incremental temperatures to 95℃ for 15 s plus 65℃ for 15 s. Complete details regarding amplification of target genes can be found at http://docs.appliedbiosystems.com/pebiodocs/04364014.pdf.

**Selection and evaluation of internal control genes (ICG)**

Data were calculated with the 7900 HT Sequence Detection Systems Software (version 2.2.1, Applied Biosystems) using a five-point standard curve, as required for the evaluation of gene stability. Stability (M = gene – stability measure) using geNorm refers to the constancy of the expression ratio between two non-co-regulated genes among all samples tested. The more stable the expression ratio among two genes, the more likely that the genes are appropriate internal controls, i.e. two ideal control genes should have an identical expression ratio in all samples regardless of experimental conditions, cell, and/or tissue type. The lower the M value, the higher the stability. geNorm also performs an analysis to determine the utility of including more than 2 genes for normalization by calculating the pairwise variation (V) between the normalization factor (NF) obtained using n genes (best references) (NF_n_) and the NF obtained using n+1 genes (addition of an extra less stable reference gene) (NF_n+1_). A large decrease in the pairwise variation indicates that addition of the subsequent more stable gene (i.e. with lowest M value) has a significant effect and should be included for calculation of the NF. Once the most stable internal reference genes are selected, the NF is calculated using the geometrical average between them to normalize qPCR data.

At the beginning, a total of seven genes were selected as internal control genes, including β-actin, β_2_ microglobulin (β-2M), Procollagen-lysine-2-oxoglutarate 5-dioxygenase 3 (PLOD3), neuroglobin (NGB), Glyceraldehyde-3-phosphate dehydrogenase (GAPDH), sentrin specific peptidase 7 (SENP7), and syntaxin 8 (STX8). According to the results generated by geNorm, β-actin, β_2_ microglobulin (β-2M), Procollagen-lysine-2-oxoglutarate 5-dioxygenase 3 (PLOD3), and neuroglobin (NGB) expression data were the four most stable genes among the ones tested as internal controls.

**Selection of genes and primer design**

Thirty-nine genes encompassing networks with central biological functions in lipid metabolism were evaluated (Figure 1). Those genes encode proteins required for fatty acid uptake, fatty acid oxidation, de novo fatty acid synthesis, lipolysis, lipogenesis, ketogenesis, carbohydrate metabolism, apoptosis, inflammation, stress response, growth factor signaling, transcriptional regulation of lipogenesis and lipid droplet formation. Primers were designed with Primer Express software fixing the amplicon length to 100-120 bp with low specific binding at the 3’-end (limit 3’-G+C; Applied Biosystems). Primers for the 39 selected genes and the 4 internal control genes are shown in Table S2. Primers were aligned against publicly available databases using BLAST software at the National Center of Biotechnology Information and also UCSC’s Mouse Genome Browser Gateway (http://genome.ucsc.edu/cgi-bin/hgGateway?hgsid=183612843&clade=mammal&org=Mouse&db=0). Prior to qPCR, primers were tested by semiquantitative PCR using a total of 20 μl mixture composed of 8 μl pooled cDNA, 10 μl SYBR Green, 1μl each of forward and reverse primers. The PCR reaction was set to 2 min at 50℃, 10 min at 95℃, 40 cycles of 15 s at 95℃ (denaturation). PCR products were run in a 3% agarose gel (Invitrogen) stained with ethidium bromide. PCR products were sequenced at the Core DNA Sequencing Facility of the Roy J. Carver Biotechnology Center at the University of Illinois, Urbana-Champaign. Aliquots of all PCR products are stored to enable additional confirmatory sequencing of PCR products in the future if required.

**Table S1.** qPCR performance among the thirty-nine genes measured in liver.

| **Gene** | **Median Ct^1^** | **Median ∆Ct^2^** | **Slope^3^** | **(R^2^)^4^** | **Efficiency^5^** |
| --- | --- | --- | --- | --- | --- |
| *Aacs* | 24.6 | 3.0 | -3.91 | 0.991 | 1.80 |
| *Acaca* | 24.4 | 2.8 | -3.39 | 0.997 | 1.97 |
| *Acox1* | 20.6 | -1.1 | -3.54 | 0.996 | 1.92 |
| *Angptl3* | 22.7 | 1.0 | -2.78 | 0.988 | 2.29 |
| *Angptl4* | 28.5 | 6.8 | -3.82 | 0.948 | 1.83 |
| *Apob* | 19.9 | -1.7 | -4.01 | 0.976 | 1.78 |
| *Atf6* | 26.3 | 4.6 | -3.07 | 0.971 | 2.12 |
| *Bdh1* | 21.7 | 0.0 | -3.58 | 0.997 | 1.90 |
| *Cd36* | 26.5 | 4.9 | -3.27 | 0.906 | 2.02 |
| *Chrebp* | 21.9 | 0.3 | -3.72 | 0.995 | 1.86 |
| *Cideb* | 21.6 | 0.0 | -2.95 | 0.999 | 2.18 |
| *Cpt1a* | 23.0 | 1.3 | -4.37 | 0.971 | 1.69 |
| *Ddit3* | 26.6 | 5.0 | -2.80 | 0.992 | 2.28 |
| *Dgat1* | 25.7 | 3.9 | -2.93 | 0.992 | 2.19 |
| *Dgat2* | 21.1 | -0.5 | -3.20 | 0.993 | 2.05 |
| *Eif2ak3* | 26.2 | 4.5 | -2.13 | 0.981 | 2.94 |
| *Fgfr2* | 26.7 | 5.2 | -2.86 | 0.974 | 2.24 |
| *Gck* | 24.1 | 2.5 | -4.03 | 0.990 | 1.77 |
| *Gpam* | 21.7 | 0.1 | -3.63 | 0.997 | 1.89 |
| *Hngcs2* | 19.5 | -2.1 | -3.47 | 0.998 | 1.94 |
| *Hspa1b* | 25.6 | 3.9 | -2.62 | 0.980 | 2.41 |
| *Insig1* | 21.4 | -0.2 | -2.55 | 0.997 | 2.47 |
| *Lipc* | 21.5 | -0.2 | -2.26 | 0.981 | 2.77 |
| *Mlycd* | 24.5 | 2.8 | -3.44 | 0.998 | 1.95 |
| *Mttp* | 23.2 | 1.5 | -3.46 | 0.999 | 1.95 |
| *Pc* | 23.1 | 1.4 | -3.87 | 0.995 | 1.81 |
| *Pck1* | 20.0 | -1.8 | -2.86 | 0.999 | 2.24 |
| *Pdk4* | 26.2 | 4.5 | -3.20 | 0.991 | 2.05 |
| *Plin2* | 22.7 | 1.1 | -3.39 | 0.996 | 1.97 |
| *Pnpla2* | 25.1 | 3.2 | -3.20 | 0.994 | 2.05 |
| *Ppara* | 23.3 | 1.6 | -3.84 | 0.996 | 1.82 |
| *Saa1* | 23.9 | 2.2 | -2.79 | 0.997 | 2.28 |
| *Scd* | 22.0 | 0.4 | -3.36 | 0.963 | 1.99 |
| *Slc27a2* | 21.5 | -0.1 | -3.99 | 0.997 | 1.78 |
| *Slc2a1* | 27.3 | 5.6 | -3.33 | 0.987 | 2.00 |
| *Srebf1* | 23.9 | 2.2 | -3.23 | 0.997 | 2.04 |
| *Ucp2* | 25.8 | 4.1 | -3.27 | 0.993 | 2.02 |
| *Xbp1* | 21.0 | -0.7 | -3.57 | 0.959 | 1.91 |

^1^ The median is calculated considering all time points and all steers.

^2^ The median of ∆Ct is calculated as [Ct gene – geometrical mean of Ct internal controls] for each time point and each steer.

^3^ Slope of the standard curve.

^4^ R2 stands for the coefficient of determination of the standard curve.

^5^ Efficiency is calculated as [10^(-1 / Slope)^].

Table S2. Accession number, sequence, amplicon size, reference and PCR efficiency of primers use to analyze gene expression by qPCR.

| **Accession #** | **Gene** | **Primer** | **Primers (5’-3’)** | **Amplicon Size (bp)** | **Source** |
| --- | --- | --- | --- | --- | --- |
| [NM_030210.1](http://www.ncbi.nlm.nih.gov/mapview/maps.cgi?maps=blast_set&db=allcontig_and_rna&na=1&gnl=ref|NM_030210.1|&gi=21313519&term=21313519%5Bgi%5D&taxid=10090&RID=N4DHDVTB01N&QUERY_NUMBER=1&log$=nucltop) | *Aacs* | F.230  R.329 | TGGCGCTTGGGAATTACAAT  CGTGAGTAGACGATTCCACTGAA | 100 | In this article |
| NM_133360.2 | *Acaca* | F.2003  R.2087 | CCTTCCTGCTCACACACTTCTG  CTGCCGAGTCACCTTAAGTACATATT | 85 |  |
| NM_015729 | *Acox1* | F.436  R.537 | GCCGTCGAGAAATCGAGAACT  TCTTAACAGCCACCTCGTAACG | 102 |  |
| NM_013913.3 | *Angptl3* |  | AGGGCTTTGGAGGAGCAGCTAACC  GCAGTCGGCAGGAAGGTCATCTTG |  |  |
| NM_020581.1 | *Angptl4* |  | TTCCAACGCCACCCACTTACA  ACCAAACCACCAGCCACCAGA |  |  |
| [NM_009693.2](http://www.ncbi.nlm.nih.gov/entrez/query.fcgi?cmd=Search&db=Nucleotide&term=NM_009693&doptcmdl=GenBank&tool=genome.ucsc.edu) | *Apob* |  | ATCTGACTGGGAGAGACAAGTAG  CGATCAATAATCTCAGCAATAGTTC |  |  |
| NM_001081304.1 | *Atf6* |  | TGCTAGGACTGGAGGCCAGGCTCAA  CATGTCTATGAACCCAGCCTCGAAGT |  |  |
| [NM_175177.3](http://www.ncbi.nlm.nih.gov/mapview/maps.cgi?maps=blast_set&db=allcontig_and_rna&na=1&gnl=ref|NM_175177.3|&gi=31982168&term=31982168%5Bgi%5D&taxid=10090&RID=N4E2XWE201S&QUERY_NUMBER=1&log$=nucltop) | *Bdh1* | F.402  R.201 | GTCAGGCAGATGCGGCTAGT  AGGAAGCCTTTTGAGTGCAGAT | 100 | In this article |
| NM_007643.3 | *Cd36* | F.726  R.846 | GAGGTCCTTACACATACAGAGTTCGTT  ACAGACAGTGAAGGCTCAAAGATG | 121 | In this article |
| NM_021455.3 | *Chrebp* | F.733  R.847 | GCTTCTAGACCTGGACTGCTTCTT  AGCATTGCCAACATAAGCATCTT | 115 |  |
| NM_009894.2 | *Cideb* |  | CCCCAAGAGTGGGATGTTGT  GGGATTTTGCTTGTACACATCGA | 100 | In this article |

| Table S2 (cont.) |  |  |  |  |  |
| --- | --- | --- | --- | --- | --- |
| **Accession #** | **Gene** | **Primer** | **Primers (5’-3’)** | **Amplicon Size (bp)** | **Source** |
| NM_013495.1 | *Cpt1a* | F.1722  R.1838 | CCTGCATTCCTTCCCATTTG  TTGCCCATGTCCTTGTAATGTG | 117 |  |
| [NM_007837.2](http://www.ncbi.nlm.nih.gov/entrez/query.fcgi?cmd=Retrieve&db=Nucleotide&list_uids=124339828&dopt=GenBank&RID=XEPZ347H01N&log$=nucltop&blast_rank=1) | *Ddit3* |  | GCAAGGAAGAACTAGGAAACGGA  CAATGTACCGTCTATGTGCAAGC |  |  |
| [NM_010046.2](http://www.ncbi.nlm.nih.gov/mapview/maps.cgi?maps=blast_set&db=allcontig_and_rna&na=1&gnl=ref|NM_010046.2|&gi=31981804&term=31981804%5Bgi%5D&taxid=10090&RID=N9EUKND7013&QUERY_NUMBER=1&log$=nucltop) | *Dgat1* | F.804  R.923 | ACAACCTGACCTACCGAGATCTCT  TCTCAAGAACTCGTCGTAGCAGAA | 120 | In this article |
| [NM_026384.3](http://www.ncbi.nlm.nih.gov/mapview/maps.cgi?maps=blast_set&db=allcontig_and_rna&na=1&gnl=ref|NM_026384.3|&gi=118129808&term=118129808%5Bgi%5D&taxid=10090&RID=N9F3BFV001S&QUERY_NUMBER=1&log$=nucltop) | *Dgat2* | F.551  R.656 | CCAAGAAAGGTGGCAGGAGAT  GTCAGCAGGTTGTGTGTCTTCAC | 106 | In this article |
| NM_010121.2 | *Eif2ak3* |  | AACGGAAGGAGTCTGAAACTCAGT  TTGGCTCAAAATCTGTTAGGTATCG |  |  |
| NM_201601.2 | *Fgfr2* |  | AAGGTTTACAGCGATGCCCA  ACCACCATGCAGGCGATTAA |  |  |
| [NM_010292.4](http://www.ncbi.nlm.nih.gov/mapview/maps.cgi?maps=blast_set&db=allcontig_and_rna&na=1&gnl=ref|NM_010292.4|&gi=118129970&term=118129970%5Bgi%5D&taxid=10090&RID=N4FE0JGH01N&QUERY_NUMBER=1&log$=nucltop) | *Gck* | F.389  R.489 | TCTCTGACTTCCTGGACAAGCA  GATGCCCTTGTCTATGTCTTCGT | 101 | In this article |
| [NM_008149.3](http://www.ncbi.nlm.nih.gov/mapview/maps.cgi?maps=blast_set&db=allcontig_and_rna&na=1&gnl=ref|NM_008149.3|&gi=141802122&term=141802122%5Bgi%5D&taxid=10090&RID=N9FTHH7C01S&QUERY_NUMBER=1&log$=nucltop) | *Gpam* | F.725  R.824 | TGGGTGTTACTAAAGCTCTTCAACA  GCGGCAGGTTCGTCTCAGT | 100 | In this article |
| NM_008256.3 | *Hmgcs2* | F.515  R.621 | CACTGACATCGAGGGCATAGATAC  CATAGCGACCATCCCAGTAGCT | 107 | In this article |
| [NM_010478.2](http://www.ncbi.nlm.nih.gov/entrez/query.fcgi?cmd=Retrieve&db=Nucleotide&list_uids=124339825&dopt=GenBank&RID=XER1Y9ZF01N&log$=nucltop&blast_rank=1) | *Hspa1b* |  | AATTTAACAGTCAACGCAATTACC  AACAGACTCTTTGCACTTGATAGC |  |  |
| NM_153526.3 | *Insig1* |  | AGGACGACAGTTAGCTATGGGTG  CCCCCTTACCCGACTCTCACATAC |  |  |

| Table S2 (cont.) |  |  |  |  |  |
| --- | --- | --- | --- | --- | --- |
| **Accession #** | **Gene** | **Primer** | **Primers (5’-3’)** | **Amplicon Size (bp)** | **Source** |
| [NM_008280.2](http://www.ncbi.nlm.nih.gov/entrez/query.fcgi?cmd=Retrieve&db=Nucleotide&list_uids=31982277&dopt=GenBank&RID=WYW2J0V301N&log$=nucltop&blast_rank=1) | *Lipc* | F.722  R.831 | TTCTCGGAGCAAAGTTCACCTAA  CCAGCCCTGTGATTCTTCCA | 110 | In this article |
| [NM_019966.2](http://www.ncbi.nlm.nih.gov/mapview/maps.cgi?maps=blast_set&db=allcontig_and_rna&na=1&gnl=ref|NM_019966.2|&gi=56797738&term=56797738%5Bgi%5D&taxid=10090&RID=N4CP269201S&QUERY_NUMBER=1&log$=nucltop) | *Mlycd* | F.903  R.1007 | CGCTGCCATCTTCTACTCCAT  AATTCCTTCTGCAGCTCCTTGA | 105 | In this article |
| [NM_008642.1](http://www.ncbi.nlm.nih.gov/mapview/maps.cgi?maps=blast_set&db=allcontig_and_rna&na=1&gnl=ref|NM_008642.1|&gi=6678959&term=6678959%5Bgi%5D&taxid=10090&RID=N9E6P12901N&QUERY_NUMBER=1&log$=nucltop) | *Mttp* | F.234  R.334 | GACAGCGTGGGCTACAAAATC  GCTGTTATCGTGACTTGGATCACTT | 101 | In this article |
| [NM_008797.2](http://www.ncbi.nlm.nih.gov/mapview/maps.cgi?maps=blast_set&db=allcontig_and_rna&na=1&gnl=ref|NM_008797.2|&gi=142351600&term=142351600%5Bgi%5D&taxid=10090&RID=N9CNRTP2013&QUERY_NUMBER=1&log$=nucltop) | *Pc* | F.686  R.799 | CGCATGAGTTCTCCAACACCTA  TGTAATTCTCTTCCAACTCCTCATAGC | 114 | In this article |
| [NM_011044.2](http://www.ncbi.nlm.nih.gov/entrez/query.fcgi?cmd=Retrieve&db=Nucleotide&list_uids=118130217&dopt=GenBank&RID=X409VHW9014&log$=nucltop&blast_rank=1) | *Pck1* | F.1453  R.1576 | CTGAAGGTGTCCCCCTTGTC  GGGTCGTGCATGATGATCTTG | 124 | In this article |
| [NM_013743.2](http://www.ncbi.nlm.nih.gov/entrez/query.fcgi?cmd=Retrieve&db=Nucleotide&list_uids=118130875&dopt=GenBank&RID=XES4JWGR012&log$=nucltop&blast_rank=1) | *Pdk4* |  | CACATGCTCTTCGAACTCTTCAAG  TGATTGTAAGGTCTTCTTTTCCCAAG |  |  |
| [NM_007408.3](http://www.ncbi.nlm.nih.gov/mapview/maps.cgi?maps=blast_set&db=allcontig_and_rna&na=1&gnl=ref|NM_007408.3|&gi=116235488&term=116235488%5Bgi%5D&taxid=10090&RID=N9NEF635013&QUERY_NUMBER=1&log$=nucltop) | *Plin2* | F.725  R.825 | AGCTGGAGATGGAAGCAAAAAA  CCGAGAGCAGAGCTTGGTAGA | 101 | In this article |
| NM_025802.2 | *Pnpla2* | F.875  R.995 | TGGAACCAAAGGACCTGATGAC  AACAAGCGGATGGTGAAGGA | 121 | In this article |
| NM_011144.3 | *Ppara* |  | ACTTCGCTATCCAGGCAGAA  CAGACCAACCAAGTGTTGTGA | 117 |  |
| [NM_009117.3](http://www.ncbi.nlm.nih.gov/entrez/query.fcgi?cmd=Retrieve&db=Nucleotide&list_uids=118130531&dopt=GenBank&RID=XEDHMCJZ01S&log$=nucltop&blast_rank=1) | *Saa1* |  | GAAGGAAGCTAACTGGAAAAACTC  CAGGCCCCCAGCACAACCTACT |  |  |

| Table S2 (cont.) |  |  |  |  |  |
| --- | --- | --- | --- | --- | --- |
| **Accession #** | **Gene** | **Primer** | **Primers (5’-3’)** | **Amplicon Size (bp)** | **Source** |
| NM_009127.3 | *Scd* |  | TCCAGTGAGGTGGTGTGAAA  TTATCTCTGGGGTGGGTTTG |  |  |
| NM_011978.2 | *Slc27a2* | F.1095  R.1194 | ACACCGCAGAAACCAAATGAC  CCCCAAATCTCTTGATGAACTCTCT | 100 | In this article |
| [NM_011400.2](http://www.ncbi.nlm.nih.gov/mapview/maps.cgi?maps=blast_set&db=allcontig_and_rna&na=1&gnl=ref|NM_011400.2|&gi=141802497&term=141802497%5Bgi%5D&taxid=10090&RID=N4F5HHYX01S&QUERY_NUMBER=1&log$=nucltop) | *Slc2a1* | F.318  R.419 | CCAGAAGGTTATTGAGGAGTTCTACAA  GCCACGGAGAGAGACCAAAG | 102 | In this article |
| NM_011480.2 | *Srebf1* |  | GTGAGCCTGACAAGCAATCA  GGTGCCTACAGAGCAAGAGG |  |  |
| NM_011671 | *Ucp2* | F.881  R.983 | GGCCTCTGGAAAGGGACTTC  TGGCTTTCAGGAGAGTATCTTTGA | 103 |  |
| NM_013842.2 | *Xbp1* |  | TCCGCAGCACTCAGACTATGT  ATGCCCAAAAGGATATCAGACTC |  |  |

Table S3. Sequencing results of PCR products from primers of genes designed for this experiment. Best hits using BLASTN (http://www.ncbi.nlm.nih.gov) are shown. Similar information for remaining genes was reported previously.

| **Gene** | **Sequence** |
| --- | --- |
| *Aacs* | GGACTTTTGGGCTGAGTTCTGGAAGTTCAGTGGAATCGTCTACTCACG |
| *Acaca* | GTACATATCTTAGTAAGGAATCAAATATGTACTTAAGGTGACTCGGCAGA |
| *Acox1* |  |
| *Angptl3* | CTCAGGAGCACCCAGAAGTAACATCACTCAAAAGTTTTGTAGAACAGCAAGACAACAGCATAAGAGAACTCCTCCAGAGTGTGGAAGAACAGTATAAACAATTAAGTCAACAGCACATGCAGATAAAAGAAATAGAAAAGCAGCTCAGAAAGACTGGTATTCAAGAACCCTCAGAAAATTCTCTTTCTTCTAAATCAAGAGCACCAAGAACTACTCCCCCTCTTCAACTGAACGAAACAGAAAATACAGAACAAGATGACCTTCCTGCCGACTGC |
| *Angptl4* | GGGAGCGGCACAGTGGACTTTTCCAGATCCAGCCTCTGGGGTCTCCACCATTTTTGGTCAACTGTGAGATGACTTCAGATGGAGGCTGGACAGTGATTCAGAGACGCCTGAACGGCTCTGTGGACTTCAACCAGTCCTGGGAAGCCTACAAGGATGGCTTCGGAGATCCCCAAGGCGAGTTCTGGCTGGGCCTGGAAAAGATGCACAGCATCACAGGGAACCGAGGAAGCCAATTGGCTGTGCAGCTCCAGGACTGGGATGGCANTGCCNAATTGCTCCAATTTCCCATCCATTTGGGGGGTGAGGA |
| *Apob* | AAATTATAGAATTACAGATAATGATGTACTAATTGCCATAGATAGTGCCAAAATCAACTTCAATGAAAAACTCTCTCAACTTGAGACATACGCGATATAATTTGATCAGTATATTAAAGATAATTATGATCCACATGACTTAAAAAGAACTATTGCTGAGATTATTGATCGAA |
| *Atf6* | AGGAGAATGGCTCCCTGAAGCGACAGCTGGACGAGGTGGTGTCAGAGAACCAGAGGCTCAAAGTCCCAAGTCCAAAGCGAAGAGCTGTCTGTGTGATGATAGTATTAGCATTTATAATGCTGAACTATGGGCCCATGAGCATGCTGGAGCAAGAATCCCGAAGAGTGAAACCTAGTGTGAGCCCTGCCAATCAGAGGAGGCATCTCTTGGAATTTTCAGCAAAAGAAGTTAAAGACACATCAGATGGTGACAACCAGAAAGACAGTTACAGCTATGATCACTCTGTGTCCAATGACAAAGCTTTAATGGTGCTAAGTGAAGAGCCATTGCTTTATATGCCTCCACCTCCATGTCAACCCCTGATTAACACAACAGAGTCTCTCAGGTTGAACCATGAACTTCGAGGCTGGGTTCATAGACATGA |

| Table S3 (cont.) | |
| --- | --- |
| **Gene** | **Sequence** |
| *Bdh1* | GATTTGGGTTCTCACTGGCCAAGCATCTGCACTCAAAAGGCTTCCT |
| *Cd36* | CCATCTTTGAGCCTTCACTGTCTGT |
| *Chrebp* | CTCTTCACCATGACACAGCCCAGTCCTTCGTCCCTGCAGCTGCCCCCAGAAGAATGCTTATGTTGGCAATGCTAA |
| *Cideb* | AGCAAGGACATCGCCCGCATCACCTTCGATGTGTACAAGCAAAATCCCC |
| *Cpt1a* | CTGATCAGAAGTGCCGGACGAGTCCCGATGCCTTCATCCAGCTGGCACTGCAGCTCGCACATTACAAGGACATGGGCAAA |
| *Ddit3* | CCGGCCTGGGAGCACGCATGAAGGAGAAGGAGCAGGAGAACGAGCGGAAAGTGGCACAGCTAGCTGAAGAGAACGAGCGGCTCAAGCAGGAAATCGAGCGCCTGACCAGGGAGGTGGAGACCACACGGCGGGCTCTGATCGACCGCATGGTCAGCCTGCACCAAGCATGAACAGTGGGCATCACCTCCTGTCTGTCTCTCCGGAAGTGTACCCAGCACCATCGCGCCAGCGCCAAGCATGTGACCCTGCACTGCACTGCACATGCTGAGGAGGGGACTGAGGGTAGACCAGGAGAGGGCTCGGCTTGCACATAGACGGTACATTG |
| *Dgat1* | TCAACTTTCCTCGGTCCCCCCGAATACGAAAGCGCTTTCTGCTACGACGAGTTCTTGAGA |
| *Dgat2* | GTGTGCGGCTACTTCCGAGACTACTTTCCCATCCAGCTGGTGAAGACACACAACCTGCTGAC |
| *Eif2ak3* | GTGCCGATGTCAGTGACAACAGCTGGAATGACATGAAGTACTCAGGATACGTATCCCGATACCTAACAGATTTTGAGCCAA |
| *Fgfr2* | ACGGCAGTAAATACGGGCCTGATGGGCTGCCCTACCTCAAGGTCCTGAAGCACTCGGGGATAAATAGCTCCAATGCAGAAGTGCTGGCTCTGTTCAATGTGACGGAGATGGATGCTGGGGCAATATATATGTAAGGTCTCCAATTATATAGGGCAGGCCAACCAGTCTGCCTGGCTCACTGTCCTGCCCAAACAGCAAGCGCCTGTGAGAGAGAAGGAGATCACGGCTTCCCCAGATTATCTGGAGATAGCTATTTACTGCATAGGGGTCTTCTTAATCGCCTGCATGGTG |
| *Gck* | CCCCGTGGCTTCACCTTCTCCTTCCCGTTAAGGCACGAAGACATAGACAAGGGCATC |
| *Gpam* | AGGGTCAACTCGAGATGGTCAAGGCTGCAACTGAGACGAACCTGCCGC |
| *Hmgcs2* | GCGGCACAGCCTCCCTCTTCAATGCTGCCAACTGGATGGAGTCCAGCTACTGGGATGGTCGCTATG |

| Table S3 (cont.) | |
| --- | --- |
| **Gene** | **Sequence** |
| *Hspa1b* | CCATGAAGAAGACTTTAAATAACCTTGACAGTAATCGGTGCCCAAGCAGCTATCAAGTGCAAAGAGTCTGTT |
| *Insig1* | CACATGGTTCAGAGTGGAAGCAGGATGTAGAGACACTGGTCCTGGGTGTGATGAAGATGATCTTTTTCTCAATGTTCCATTTAGGCTGGGCTGACGATAAATGACTCCTAAAGACATGTTCACTTAGTCTAGACTAGCAAATGGAGGCAAGGACTGCTTACCTAAAAGTCTTACCTTGCTCCCCCACCCTCACACCTGTCTTCTTTGGAACATTCCATCCCAGGCTGTATGTGAGAGTCGGGGTAAGGGGG |
| *Lipc* | CGTCTCAGGGTTCGCAGGCAGCTCCATGGACGGGAAGAACAAGATTGGAAGAATCACAGGGC |
| *Mlycd* | GAGCTCGGGACCTTCCTCATAAAGAGAGTGGTCAAGGAGCTGCAGAAGGAATT |
| *Mttp* | GGAGGAATCCTGATGGTGATGATGATCAAGTGATCCAAGTCACGATAACAGC |
| *Pc* | GCCGCCTACGGAGGTGGGGGCCGCGGCATGCGGGTCGTGCATAGCTATGAGGAGTTGGAAGAGAATTACA |
| *Pck1* | GGAGCAGCCATGAGATCTGAGGCCACAGCTGCTGCAGAACACAAGGGCAAGATCATCATGCACGACCC |
| *Pdk4* | GAGCATCAAGAAAACCGTCCTTCCTTGACCCCAGTAGAGGCCACTGTCGTCTTGGGAAAAGAAGACCTTACAATCAA |
| *Plin2* | AAGCCGAGCAACTATGAACGGCTGGAGTCCCTGTCTACCAAGCTCTGCTCTCGGG |
| *Plin5* | CCCCAAAGACTCAGGAGCTGTGGGGGAGCTGGAGCCCGTGTCTAGAGAATGGCCGCAGC |
| *Pnpla2* | GCGCCTGGCAACGGCCATGATGGTGCCCTATACTCTGCCGCTGGAGAGTGCAGTGTCCTTCACCATCCGCTTGTT |
| *Ppara* |  |
| *Saa1* | ATGCTGCTCAAAGGGGTCCCGGGGGAGTCTGGGCTGCTGAGAAAATCAGTGATGGAAGAGAGGCCTTTCAGGAATTCTTCGGCAGAGGACATGAGGACACCATTGCTGACCAGGAAGCCAACAGACATGGCCGCAGTGGCAAAGACCCCAATTACTACAGACCTCCTGGACTGCCTGACAAATACTGAGCGTCCTCCTATTAGCTCAGTAGGTTGTGCTGGGGGCCTGA |
| *Scd* | CTCAGCCAAGGTGCCTCTTAGCCACTGAATTGCTATGTTATCCTTTCTCTTGTAACAAACCCACCCCAGAGATAAA |
| *Slc27a2* | GGAAATGGCTTACGAGGAGATGTGTGGAGAGAGTTCATCAAGAGATTTGGGG |

| Table S3 (cont.) | |
| --- | --- |
| **Gene** | **Sequence** |
| *Slc2a1* | GGAGAGCCCATCCCATCCACCACACTCACCACGCTTTGGTCTCTCTCCGTGGC |
| *Srebf1* | GGTCAAGAATCTGGAAATTGCAGAGGCTGCACTGGCCCGATGGCACCCTCTTGCTCTGTAGGCACCAAT |
| *Ucp2* |  |
| *Xbp1* | TCCCCAGAACATCTTCCCATGGACTCTGACACTGTTGCCTCTTCAGATTCTGAGTCTGATATCCTTTTGGGCAT |

Table S4. Sequencing results of genes using BLASTN from NCBI against nucleotide collection (nr / nt) with total score.

| **Gene** | **Best hit in NCBI** | **Score** |
| --- | --- | --- |
| *Aacs* | Mus musculus acetoacetyl-CoA synthetase (Aacs), mRNA | 80.1 |
| *Acaca* | Mus musculus acetyl-Coenzyme A carboxylase alpha (Acaca), mRNA | 89.1 |
| *Acox1* | Mus musculus acyl-Coenzyme A oxidase 1, palmitoyl (Acox1), mRNA. | 80.1 |
| *Angptl3* | Mus musculus angiopoietin-like 3 (Angptl3), mRNA | 89.1 |
| *Angptl4* | Mus musculus angiopoietin-like 4 (Angptl4), mRNA | 78.3 |
| *Apob* | Mus musculus chromosome 12 genomic contig, strain C57BL/6J | 89.1 |
| *Atf6* | Mus musculus activating transcription factor 6 (Atf6), mRNA | 94.5 |
| *Bdh1* | Mus musculus 3-hydroxybutyrate dehydrogenase, type 1 (Bdh1),mRNA | 78.3 |
| *Cd36* | Mus musculus CD36 antigen (Cd36), mRNA | 94.5 |
| *Chrebp* | Mus musculus MLX interacting protein-like (Mlxipl), Mrna | 87.3 |
| *Cideb* | Mus musculus cell death-inducing DNA fragmentation factor, alpha subunit-like effector B (Cideb), mRNA | 80.1 |
| *Cpt1a* | Mus musculus carnitine palmitoyltransferase 1a, liver (Cpt1a), | 78.3 |
| *Ddit3* | Mus musculus DNA-damage inducible transcript 3 (Ddit3), mRNA | 85.5 |
| *Dgat1* | Mus musculus diacylglycerol O-acyltransferase 1 (Dgat1), mRNA | 89.1 |
| *Dgat2* | Mus musculus diacylglycerol O-acyltransferase 2 (Dgat2), mRNA | 81.9 |
| *Eif2ak3* | Mus musculus eukaryotic translation initiation factor 2 alpha kinase 3 (Eif2ak3), mRNA | 81.9 |
| *Fgfr2* | Mus musculus fibroblast growth factor receptor 2 (Fgfr2), transcript variant 2, mRNA | 74.7 |
| *Gck* | Mus musculus glucokinase (Gck), mRNA | 83.7 |
| *Gpam* | Mus musculus glycerol-3-phosphate acyltransferase, mitochondrial (Gpam), mRNA | 81.9 |
| *Hmgcs2* | Mus musculus 3-hydroxy-3-methylglutaryl-Coenzyme A synthase 2 (Hmgcs2), mRNA | 85.5 |
| *Hspa1b* | Mus musculus heat shock protein 1B (Hspa1b), mRNA | 89.1 |
| *Insig1* | Mus musculus insulin induced gene 1 (Insig1), mRNA | 87.3 |

| Table S4 (cont.) | | |
| --- | --- | --- |
| **Gene** | **Best hit in NCBI** | **Score** |
| *Lipc* | Mus musculus lipase, hepatic (Lipc), mRNA | 80.1 |
| *Mlycd* | Mus musculus malonyl-CoA decarboxylase (Mlycd), mRNA | 80.1 |
| *Mttp* | Mus musculus microsomal triglyceride transfer protein (Mttp), mRNA | 83.7 |
| *Pc* | Mus musculus pyruvate carboxylase (Pcx), mRNA | 89.1 |
| *Pck1* | Mus musculus phosphoenolpyruvate carboxykinase 1, cytosolic (Pck1), mRNA | 76.5 |
| *Pdk4* | Mus musculus pyruvate dehydrogenase kinase, isoenzyme 4 (Pdk4), mRNA | 92.7 |
| *Plin2* | Mus musculus adipose differentiation related protein (Adfp), mRNA | 80.1 |
| *Plin5* | Mus musculus RIKEN cDNA 2310076L09 gene (2310076L09Rik), transcript variant 1, mRNA | 76.5 |
| *Pnpla2* | Mus musculus patatin-like phospholipase domain containing 2 (Pnpla2) mRNA | 78.3 |
| *Ppara* | Mus musculus peroxisome proliferator activated receptor alpha (Ppara) | 76.5 |
| *Saa1* | Mus musculus serum amyloid A 1 (Saa1), mRNA | 85.5 |
| *Scd* | Mus musculus stearoyl-Coenzyme A desaturase 1 (Scd1), mRNA | 74.7 |
| *Slc27a2* | Mus musculus solute carrier family 27 (fatty acid transporter), member 2 (Slc27a2), mRNA | 85.5 |
| *Slc2a1* | Mus musculus solute carrier family 2 (facilitated glucose transporter),member 1 (Slc2a1), mRNA | 87.3 |
| *Srebf1* | Mus musculus sterol regulatory element binding factor 1 (Srebf1), mRNA | 74.7 |
| *Ucp2* | Mus musculus uncoupling protein 2 (mitochondrial, proton carrier) | 81.7 |
| *Xbp1* | Mus musculus X-box binding protein 1 (Xbp1), mRNA | 81.9 |

Figure S1. Evaluation of internal control genes using geNorm software.
